# The FGFR inhibitor Rogaratinib reduces microglia reactivity and synaptic loss in TBI

**DOI:** 10.3389/fimmu.2024.1443940

**Published:** 2024-11-20

**Authors:** Rida Rehman, Albrecht Froehlich, Florian olde Heuvel, Lobna Elsayed, Tobias Boeckers, Markus Huber-Lang, Cristina Morganti-Kossmann, Francesco Roselli

**Affiliations:** ^1^ Department of Neurology, Ulm University, Ulm, Germany; ^2^ Institute for Stem Cell Biology and Regenerative Medicine, Stanford University School of Medicine, Stanford, CA, United States; ^3^ German Center for Neurodegenerative Diseases (DZNE), Ulm, Germany; ^4^ Institute of Anatomy and Cell biology, Ulm University, Ulm, Germany; ^5^ Institute of Translational Trauma Immunology, Ulm University, Ulm, Germany; ^6^ Department of Child Health, Barrow Neurological Institute at Phoenix Children’s Hospital, Phoenix, AZ, United States; ^7^ University of Arizona College of Medicine, Phoenix, AZ, United States

**Keywords:** reactive microglia, traumatic brain injury, receptor tyrosine kinase, proteomics, synapses

## Abstract

**Background:**

Traumatic brain injury (TBI) induces an acute reactive state of microglia, which contribute to secondary injury processes through phagocytic activity and release of cytokines. Several receptor tyrosine kinases (RTK) are activated in microglia upon TBI, and their blockade may reduce the acute inflammation and decrease the secondary loss of neurons; thus, RTKs are potential therapeutic targets. We have previously demonstrated that several members of the Fibroblast Growth Factor Receptor (FGFR) family are transiently phosporylated upon TBI; the availability for drug repurposing of FGFR inhibitors makes worthwhile the elucidation of the role of FGFR in the acute phases of the response to TBI and the effect of FGFR inhibition.

**Methods:**

A closed, blunt, weight-drop mild TBI protocol was employed. The pan-FGFR inhibitor Rogaratinib was administered to mice 30min after the TBI and daily up to 7 days post injury. Phosphor-RTK Arrays and proteomic antibody arrays were used to determine target engagement and large-scale impact of the FGFR inhibitor. pFGFR1 and pFGFR3 immunostaining were employed for validation. As outcome parameters of the TBI injury immunostainings for NeuN, VGLUT1, VGAT at 7dpi were considered.

**Results:**

Inhibition of FGFR during TBI restricted phosphorylation of FGFR1, FGFR3, FGFR4 and ErbB4. Phosphorylation of FGFR1 and FGFR3 during TBI was traced back to Iba1+ microglia. Rogaratinib substantially dowregulated the proteomic signature of the neuroimmunological response to trauma, including the expression of CD40L, CXCR3, CCL4, CCR4, ILR6, MMP3 and OPG. Prolonged Rogaratinib treatment reduced neuronal loss upon TBI and prevented the loss of excitatory (vGLUT+) synapses.

**Conclusion:**

The FGFR family is involved in the early induction of reactive microglia in TBI. FGFR inhibition selectively prevented FGFR phosphorylation in the microglia, dampened the overall neuroimmunological response and enhanced the preservation of neuronal and synaptic integrity. Thus, FGFR inhibitors may be relevant targets for drug repurposing aimed at modulating microglial reactivity in TBI.

## Introduction

Traumatic Brain Injury (TBI) is characterized by the dynamic interplay of multiple cellular actors, including neurons, astrocytes, microglia as well as vascular and immune cells, which may assume beneficial or detrimental roles, depending on time and space (1, 2). Microglial cells swiftly react to TBI by migrating to the site of injury (3), assuming an ameboid, chemotactic morphology (4) and diverse reactive functional states including ones characterized by increased interferon response (5) and by disease-associated-like microglial markers (6, 7). Unchecked acute microglial reactivity in TBI has been largely considered detrimental, leading to acidosis, oxidative stress, enhanced neuronal damage and synaptic loss (8–10) in the “secondary injury” phase.

However, early post-traumatic depletion of microglia by CSF1R inhibitor administration reduces the extent of neuronal apoptosis but does not affect the overall lesion size and actually increased the size of intracerebral haematoma (11). Actually, a number of microglia-associated responses, such as glial limitans repair and debris clearing may have neuroprotective outcomes (12–14) and may be carried out by specific subset or functional states of microglial cells [such as repopulating microglia; (15, 16)]. Thus, the goal of suppressing microglial reactivity should be substituted by the aim for a fine-tuning microglial reactivity and phenotype to maximize tissue preservation.

Receptor tyrosine kinases have emerged in the last 20 years as a class of drug targets, with more than 70 small-molecule kinase inhibitors approved for human use [mainly in oncology; (17, 18)] and therefore lend themselves to effective drug repurposing. Notably, RKT activation is a prominent response in TBI: a targeted phosphoproteomic screening of 39 RTK has revealed the significant increase in phosphorylation of multiple families of RTK including VEGFR1-3, EphB4, Met, MSPR, EGFR, ErbB3 and FGFR4 at 3h and 24h timepoints (19). In fact, Met and VEGFR, among others, were shown to be phosphorylated in microglial cells, contributing to their regulation. Proof of concept of the use of RTK as entry points for acute TBI treatment has been provided by the use of VEGFR and Met small-molecule inhibitors: both caused the substantial divergence in the phosphoproteomic profile after TBI (demonstrating target engagement) and resulted in improved motor performance (19). Furthermore, prolonged treatment with a Met inhibitor delivered persistent improvement in motor performace and enhanced neuronal preservation (19). These findings have opened up the possibility that multiple RTKs may be involved in the early induction of reactive microglial phenotype(s) and may lend themselves to therapeutic modulation. Among these, some FGFR family members displayed transient up-phosphorylation between 3h and 24h after trauma. Interestingly, the FGFR family does not only control proliferation and plasticity, but has been implicated also in the control of inflammation: blockade of FGFRs reduce the cytokine storm and macrophage proliferation in sepsis (20) and FGFR ligand FGF23 induces TNF-α in macrophages (21). Furthermore, FGFR inhibitor Infigratinib reduced microglial rand lymphocyte responses in a multiple sclerosis murine model (22) whereas a different FGFR inhibitor reduces the inflammatory response to *B. burgdorferi* antigens (23). FGFR inhibitors appear to reduce the availability of immunoproteasome subunits by inducing autophagy in immune cells (24). Taken together, this evidence supports the hypothesis that FGFR inhibitors may modulate inflammatory responses in TBI.

Since FGFR inhibitors have been recently introduced in clinical practice (25), we set out to explore the potential role of FGFR in the early stages of the neuroinflammatory response to TBI.

## Results

### Pan-FGFR inhibitor modifies the RTK phosphorylation landscape of acute TBI

**Figure 1:**
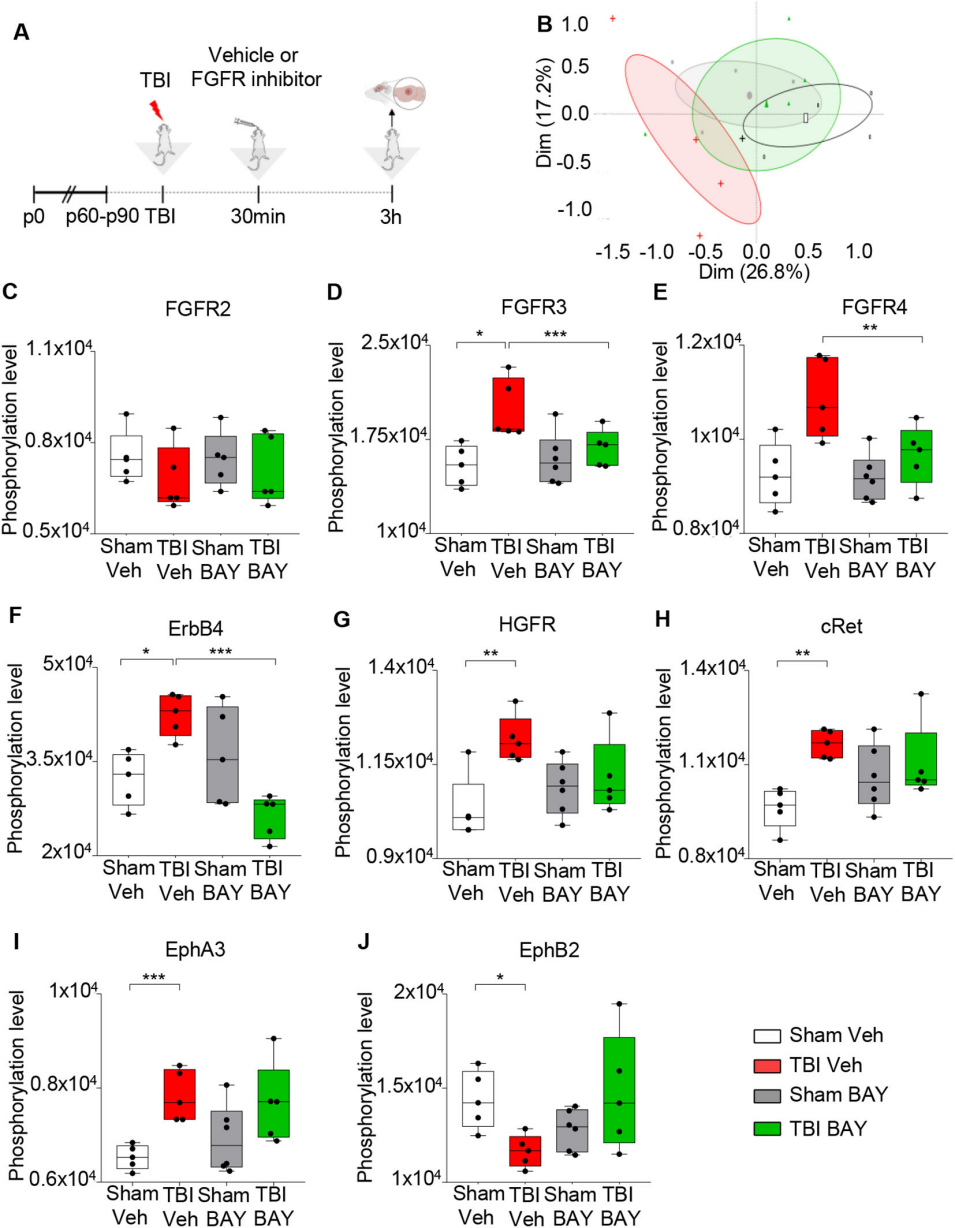
FGFR Inhibitor selectively alters RTK phosphorylation pattern at 3h post injury. **(A)** Outline of the experimental design. BAY121 (Rogaratinib) was administered by oral gavage at the dose of 25 mg/kg 30 min after TBI and samples were obtained 3h after TBI. **(B)** Principal component analysis (PCA) plot displayed group wise distribution of samples, highlighting the separation between TBI-Veh samples (red) and the BAY121-treated samples, which overlap with Sham-Veh samples. The group specific ellipses indicate 95% confidence interval. **(C-J)** Antibody phospho-array focused proteomic analysis of RTK phosphorylation patterns upon TBI with or withouth BAY121 treatment. Differential phosphorylation analysis showed the inhibitor significantly decreased the phosphorylated levels of **(D)** pFGFR3, **(E)**, pFGFR4 (the array does not include pFGFR1) and **(F)** ErbB4 after TBI. However, the inhibitor showed no effect on phosphorylation levels of **(C)** FGFR2, **(G)** HGFR, **(H)** cRET, **(I)** EphA3, and **(J)** EphB2. Significance for differentially phosphorylated proteins was set at p<0.05 (FDR adjusted). [**(B-J)**: n=5-6/group; *p<0.05, **p<0.01, ***p<0.001].

We explored the effect of the pan-FGFR inhibitor BAY 1213802 [Rogaratinib-HCl; (26); henceforth BAY121], on the TBI-associated RTK phosphorylation landscape with the goal to demonstrate effective and specific target engagement. Mice were subjected to a blunt weight-drop mild TBI (or sham surgery), followed 30 min later by administration of BAY121 (or vehicle). NSS score ranged between 0 and 1 (coherently with the mild TBI protocol) at the 3h timepoints. At 3h post injury mice were sacrificed, samples from the injury site were obtained (**Figure 1A**) and processed for RTK phosphorylation screening using a nitrocellulose antibody arrays (RTK targets including FGFR 2, 3 and 4). Principal component analysis (PCA) demonstrated a substantial overlap among S-Veh, T-BAY and S-BAY samples, whereas T-Veh stood out (**Figure 1B**). Analysis of individual RTK phosphorylation revealed a significant upregulation of p-FGFR3 and p-FGFR4 (but not of pFGFR2) upon TBI which was negated by the BAY121 treatment (**Figures 1C–E**); of note, the antibody array did not include phosphoFGFR1 antibodies. In addition, TBI upregulated the phosphorylation of HGFR, cRet, ErbB4, EphA3 and downregulated phopsho-EphB2 (**Figures 1F–J**). Interestingly, BAY121 also prevented the phosphorylation of ErbB4 (**Figure 1F**) but did not affect the up-phosphorylation of cRet and EphA3 (**Figures 1H, I**; only statistical trends were detected for HGFR nd EphB2).

Taken together, these findings demonstrate that TBI upregulates the phosphorylation of FGFR and BAY121 successfully negates this event; BAY121 (confirming target engagement) does not appear to block the phosphorylation of other RKTs with the exception of ErbB4 (supporting the selectivity of BAY121).

### FGFR inhibitor prevents FGFR1 and FGFR3 phophorylation in microglia upon trauma

**Figure 2:**
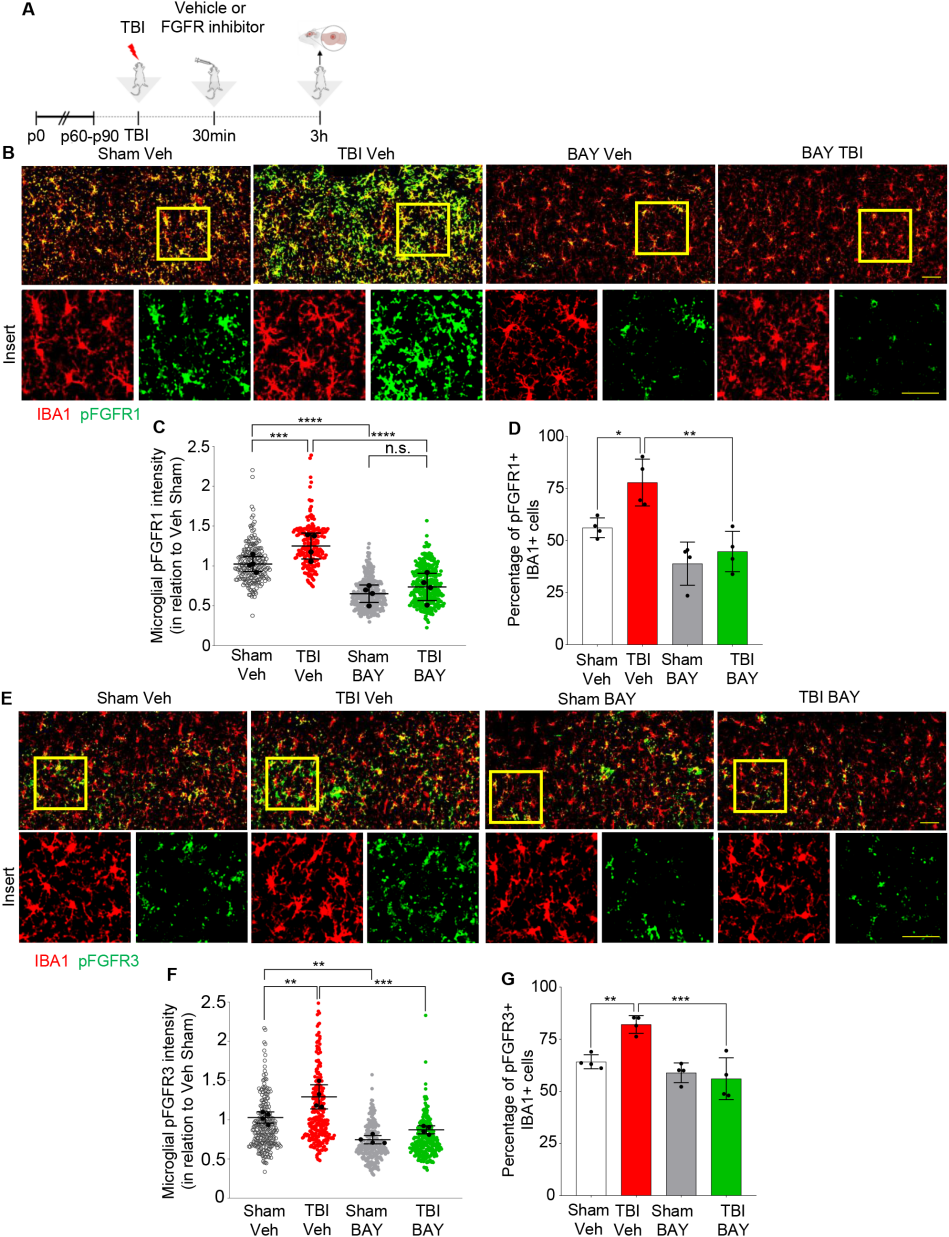
Upregulation of pFGFR1 and pFGFR3 in microglia 3h post injury. **(A)** Outline of the experimental design. BAY121 (Rogaratinib) was administered by oral gavage at the dose of 25 mg/kg 30 min after TBI and samples for immunohistology were obtained at 3h after TBI. **(B-D)** Immunostainings for pFGFR1 (green) and Iba1 (red) for Sham- Veh, TBI Veh, Sham- BAY121 and TBI BAY121 treated mice. Quantification of pFGFR1 immunostaining intensity in Iba1+ cells **(C)** and fraction of pFGFR1+ cells **(D)** display a significant increase upon TBI, which is negated by the treatment with BAY121. **(D-F)** Immunostaining for pFGFR3 (green) and Iba1 (red) shows the upregulation of pFGFR immunoreactivity in Iba1+ cells and the increase in the fraction of pFGFR3 upon TBI. Both indexes are decreased by treatment with BAY121. n=4/group; >300 cells per animal n.s., not significant; **p<0.01; ***p<0.001, ****p<0.0001. Overview Scale bar: 50µm. Inset scale bar: 20µm.

We investigated the cellular sources and the spatial distribution of FGFR phosphorylation using immunohistological approaches. Only antibodies against FGFR1(pY654) and FGFR3(pY724) proved suitable for immunolabeling of brain sections, whereas no antibody against phospho-FGFR2 and phospho-FGFR4, suitable for immunohistochemistry, was available from commercial sources; therefore, we limited our immunohistochemical study to phospho-FGFR1 and phospho-FGFR3. Animals were subject to TBI and injected 30 min later with either vehicle or BAY121 and sacrificed 3h after the injury (2.5h after treatment; **Figure 2A**). In we could detect a significant fraction of Iba1+ cells displaying pFGFR1 immunoreactivity already in S-Veh samples. Moreover, immunoreactivity for both pFGFR1 and pFGFR3 was significantly upregulated in the site of injury (“core”, located in cortical layer II/III region) in T-Veh but not in T-BAY samples (**Figures 2B, E**). Interestingly, the immunoreactivity pattern of pFGFR1 and 3 highlighted large number of small cells of ramified morphology, resembling microglia. In fact, co-immunostaining with Iba1 demonstrated that 70-90% of Iba1+ cells in the site of injury displayed immunoreactivity for pFGFR1 and >50% of Iba1+ cells displayed immunoreactivity for FGFR3. In T-Veh samples, both the immunofluorescence intensity in Iba1+ cells (**Figures 2C, F**) and the fraction of Iba1+ immunopositive for phospho FGFR1 or phospho FGFR3 was significantly increased compared to S-Veh (**Figures 2C, D, F, G**). Notably, in T-BAY samples, both the immunofluorescence intensity and the number of Iba1+ cells immunopositive for phosphoFGFR1 and FGFR3 were strongly decreased (no change in the total number of Iba1+ cells was noted).

Taken together these results confirm the elevation in FGFRs activation upon TBI and demonstrated the successful target engagement for BAY121 on microglial cells.

### FGFR inhibition significantly suppresses immune responses in the site of injury

**Figure 3:**
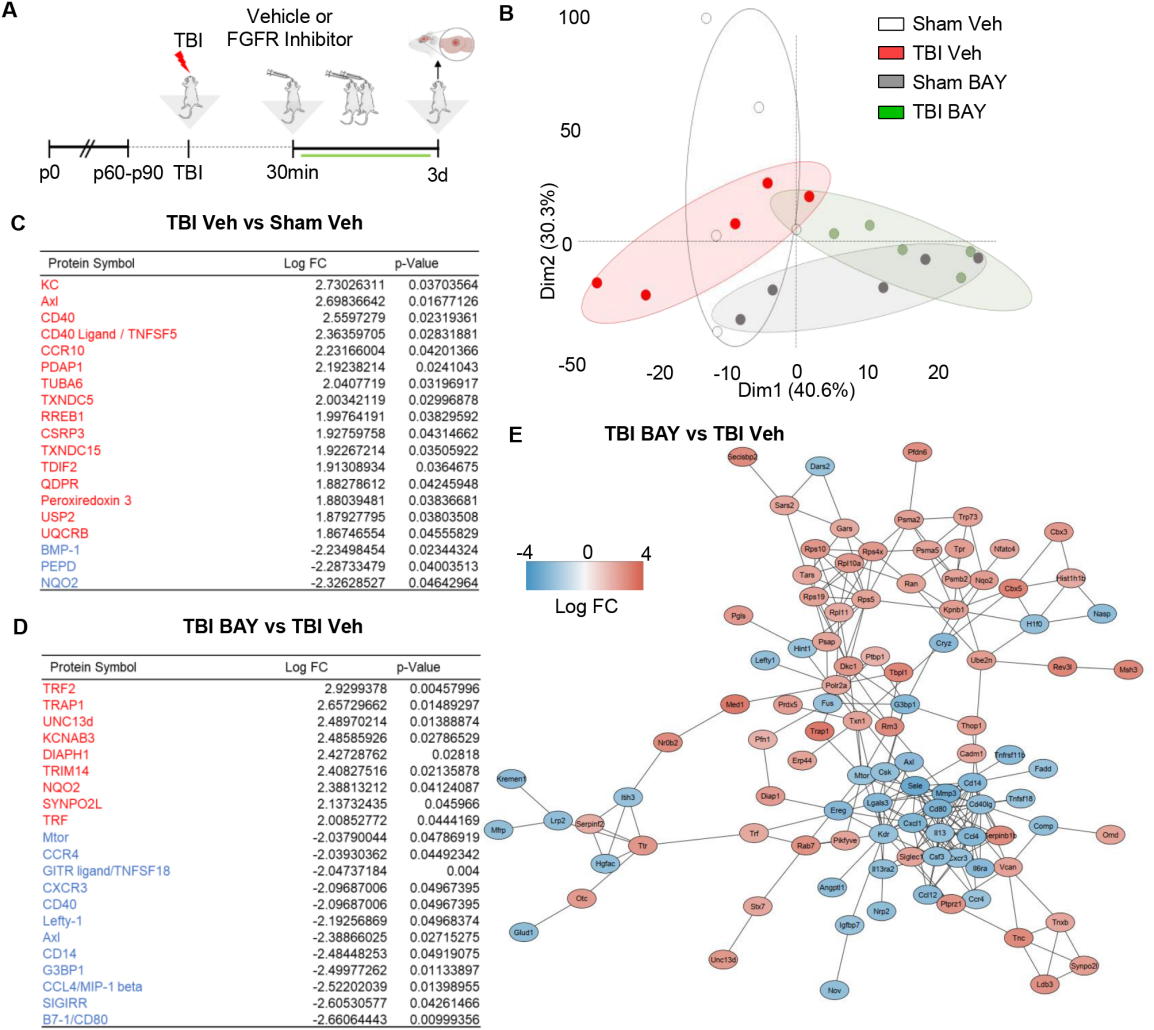
FGFR inhibitor suppresses immune-related proteome signature at 3d post injury. **(A)** Outline of the experimental design; BAY121 was administered 30 mins after trauma and continued for 3d (25 mg/Kg once daily by oral gavage, vehicle alone as control). Samples were collected 3d post injury. **(B)** PCA plot of proteomic data shows the separation of TBI-Veh from BAY121-treated samples. **(C, D)** After modified differential protein expression analysis (FDR <0.05), subsets of upregulated (Red) and downregulated (Blue) proteins with log fold change and individual significance for **(D)** TBI-Veh compared to Sham–Veh and **(D)** TBI-BAY121 compared to TBI-Veh (n=5/group). **(E)** Protein-protein-interaction analysis revealed distinct clustering of downregulated and upregulated proteins in TBI-BAY121 vs TBI Veh; the cluster of downregulated proteins is enriched with immune- and inflammation-related proteins.

Next we explored if prolonged BAY121 administration could not only affect the acute reactive microglial phenotype, but generate a long-lasting, broad alteration of the proteomic neuroimmunological landscape associated with brain injury. Mice subjected to trauma were administered with BAY121 (or vehicle) 30 mins after trauma (or sham surgery) and continued daily for 3 days (**Figure 3A**). A targeted proteomic profile of the injury site, involving >1300 individual protein was obtained by antibody arrays. PCA plot showed a substantial separation between S-Veh and TBI-Veh samples on one side and S-BAY and T-BAY on the other (**Figure 3B**). When compared to S-Veh samples, TBI-Veh samples displayed the upregulation of 16 proteins and the downregulation of 3 proteins (**Figure 3C**). The upregulated proteins involved multiple mediators of inflammatory responses, including KC (murine homologue of the chemoattractant IL-8), the microglial regulator Axl, the chemotactic receptor CCR10 and the immune regulator CD40 and its ligand CD40L. On the other hand, BAY121 resulted in a substantial change in the proteome when administered after trauma: 51/1308 proteins were downregulated and 98/1308 were upregulated in TBI-BAY121 vs TBI-Veh samples (**Figures 3D, E**, **Supplementary Figure S1A**). The gene ontology analysis revealed that BAY121 treatment resulted in the downregulation of proteins involved in immune function and cytokine response (top 5 GO categories, **Supplementary Figures S1B, C**). Strikingly, Axl and CD40L (upregulated in TBI-Veh vs Sham-Veh) were among the top strongly downregulated along with microglia polarization indicator CD80 and, proinflammatory signaling and chemotaxis markers such as CXCR3, CCL4, CCR4, ILR6, MMP3 and Osteoprotegerin [recently involved in microglial reactivity; (7)] (**Figure 3D**). The upregulated proteins display an enrichment in the GO categories of cellular signaling, metabolism and protein synthesis, including the mitochondrial protein TRAP and the ion channel KCNAb3.

We further characterized the distinct signature imposed by BAY121 treatment upon TBI by constructing a protein-protein interaction (PPI) network for the proteins up- or down-regulated by the treatment. We employed the String algorithm and visualized the network using the Cytoscape software. After preprocessing of the dataset, PPI network displayed 137 nodes and 243 edges (**Figure 3E**). Most notably, proteins up- and down-regulated by BAY121 were enriched in two distinct clusters, with most of the down-regulated proteins related to the immune response forming a tight cluster. Taken together, the proteomic data suggest that prolonged administration of the FGFR inhibitor BAY121 profoundly reduces the sub-acute neuroinflammatory response to TBI.

### Prolonged FGFR inhibitor alters both neuron-specific and immune-specific proteins 7d post injury

**Figure 4:**
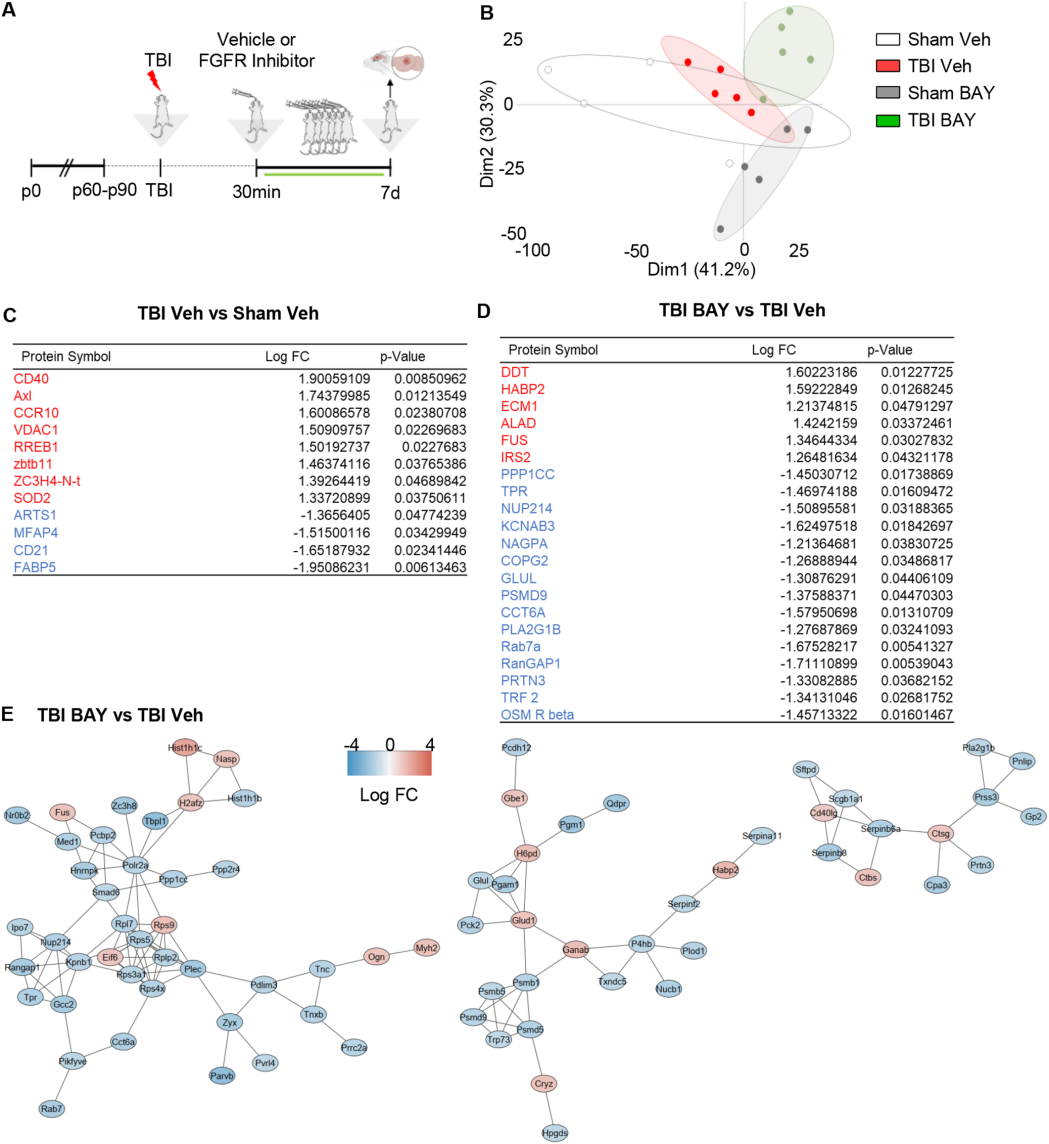
Prolonged FGFR inhibitor treatment significantly alters the TBI-related proteome profiles at 7d post injury. **(A)** Outline of the experimental design; BAY121 was administered 30 mins after trauma and continued for 7d (25 mg/Kg/day). Samples were collected 7d post injury. **(B)** PCA plot shows the substantial separation between TBI groups and groups treated with BAY121. **(C, D)** After modified differential protein expression analysis (FDR <0.05), subset of significantly upregulated (Red) and downregulated (Blue) proteins with log fold change and individual significance for **(C)** TBI-Veh vs Sham–Veh and **(D)** TBI-BAY121 vs TBI-Veh. **(E)** Protein-protein interaction analysis by STRING reveals three distinct networks affected by prolonged BAY121 treatment, related to protein synthesis proteasomal degradation and inflammation (n=5/group).

We further investigated the proteomic signature of prolonged FGFR inhibition in TBI by taking into consideration samples obtained at 7 dpi. As before, mice subjected to trauma were administered with BAY121 (or vehicle) 30 mins after trauma. The treatment was continued for 7 days (1 dose/day) for 7 days (**Figure 4A**). PCA plot based on the targeted proteomics (1308 targets), revealed that, while the S-Veh and T-Veh samples largely clustered together, S-BAY121 and TBI-BAY121 minimally overlapped the Veh groups (**Figure 4B**), indicating a persistent and profound effect of BAY121 treatment.

The analysis of differentially expressed proteins revealed the increased expression in TBI-Veh (vs Sham-Veh) of the proteins involved in inflammation such as Axl, CD40, CCR10 and SOD2, whereas the B-cell marker CD21 was downregulated (**Figure 4C**). Notably, the comparison of BAY121-treated TBI vs vehicle-treated TBI samples revealed a substantial divergence in the proteome: 123/1308 proteins were downregulated and 38/1308 proteins were upregulated (**Figures 4D, E**). The gene ontology analysis of the downregulated proteins revealed a substantial involvement of proteasome regulatory proteins and nucleo-cytoplasmic trafficking, pointing toward an impaired protein degradation and cellular stress (**Supplementary Figure S2B**). The comparatively small number of upregulated proteins did not lend itself to a reliable GO analysis.

**Figure 5:**
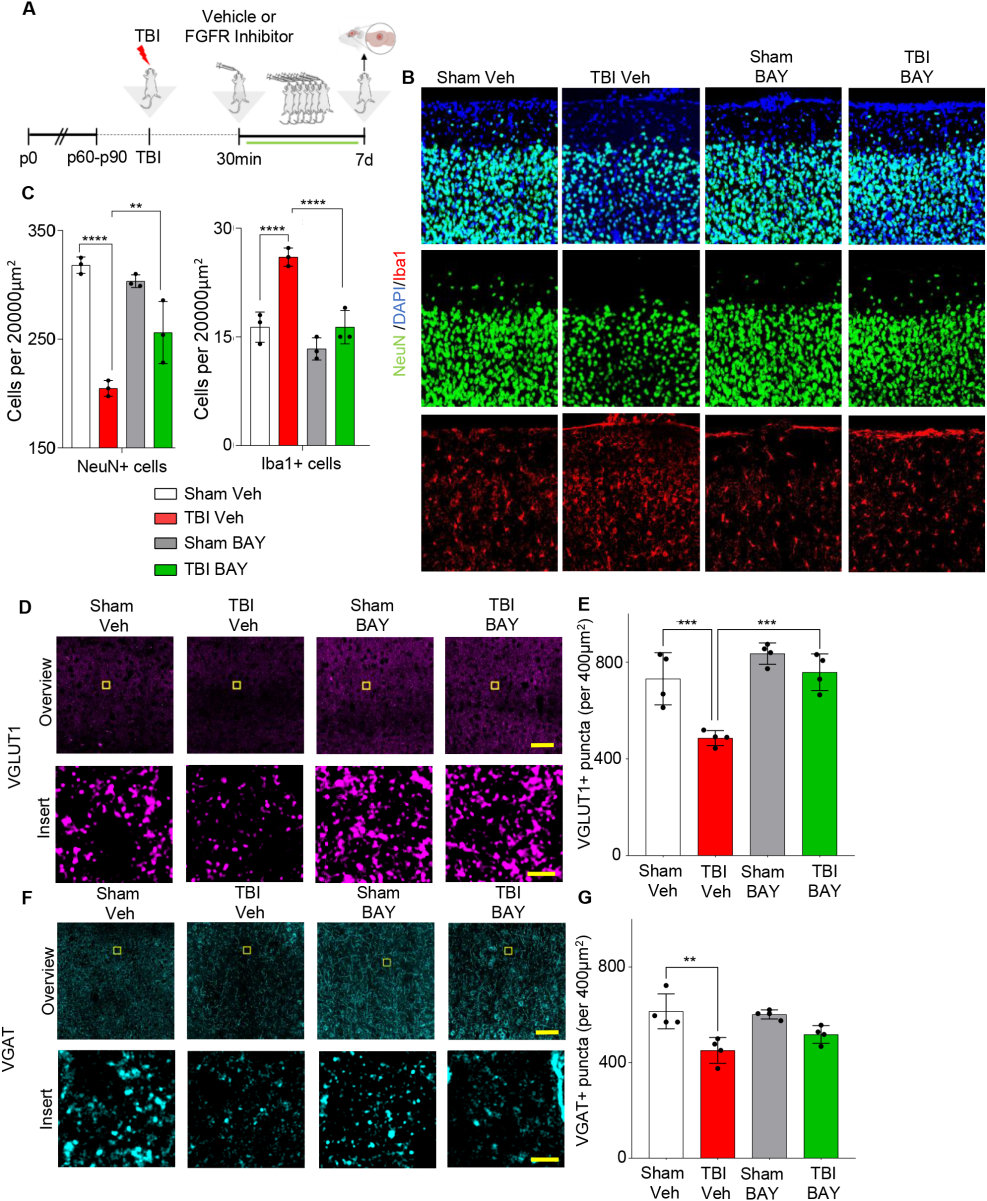
Prolonged FGFR inhibitor administration preserves neuronal density at 7 dpi. **(A)** Outline of the experimental design; BAY121 was administered 30 mins after trauma and continued for 7d (25 mg/Kg daily by oral gavage). Samples were collected 7d post injury. **(B, C)** Immunostaining for NeuN showed a significantly decreased number of neurons in the ijury site of TBI-Veh group compared to Sham–Veh; a significantly higher number of neurons was seen in the TBI-BAY121 group. Note that microglial density was also increased in TBI-Veh but not in TBI-BAY121. (n=4/group; **p<0.01, ***p<0.001, ****p<0.0001; Scalebar 70µm) **(D, E)** Loss of VGLUT1 density after TBI is dependent on FGFR signaling 7d after TBI. (n=4/group; ***p<0.001; Scale bar: overview = 70μm; insert = 5μm) **(F, G)** VGAT density in the TBI core is significantly reduced at 7dpi. (n=4/group; **p<0.01; Scale bar: overview = 70μm; insert = 5μm).

Interestingly, when we mapped the PPI of proteins altered by BAY121 treatment (TBI-BAY121 vs TBI-Veh) using STRING/Cytoscape, three distinct sub-networks (cumulatively displaying 112 nodes and 135 edges; **Figure 5E**) were identified. The smallest of the network (13 proteins) involved downregulated proteins related to the inflammatory/phagocytic function (notably Catepsins and Serpins) and immune regulation (such as CD40L). The two larger networks (65 proteins) included many subunits of the proteasome system, chaperones and trafficking proteins, the largest majority of which were downregulated (**Figure 4E**, **Supplementary Figure S2A**). Based on these findings, the proteomic analysis at 7dpi suggested that blockade of FGFR signaling produced a persistend impact on the neuroinflammatory cascade but also substantially impacted the tissue protein homeostasis.

### FGFR inhibitor reduces neuronal loss and synaptic loss 7d post trauma

Finally, we sought to determine if the effects of FGFR blockade on the neuroinflammatory response to TBI were associated with reduced neuronal vulnerability and synaptic integrity. As before, mice were subjected to trauma and treated with either vehicle or the FGFR inhibitor 30 mins after trauma for 7 days (1 dose/day) (**Figure 5A**). As expected, density of microglia was still increased in the injury site (“core”, located in layed II/III of cortex, on the axis of the site of injury) of TBI -Veh mice, but not in TBI-BAY121 group (**Figures 5B, C**). Conversely, the density of NeuN+ cells was significantly reduced in the site of injury in TBI Veh mice compared to Sham mice (**Figures 5B, C**). Notably, BAY121 treatment significant increased the number of surviving neurons in the injury site.

We further explored the preservation of synaptic structures in the injury site upon BAY121 treatment. We assessed the density (number of synaptic puncta per 400µm^2^) of excitatory synapses in the form of pre-synaptic VGLUT1 as well as of inhibitory synapses in the form of pre-synaptic VGAT. VGLUT1 showed a significant loss in synaptic density after TBI, which was not observable any more after BAY121 treatment 7dpi (**Figures 5D, E**). VGAT+ terminals were also significantly decreased in the lesion area at 7 dpi (**Figures 5F, G**) with a trend toward better preservation in T-BAY samples.

## Discussion

Our data show that FGFR family is prominently activated in the early phases of mild TBI in particular in microglial cells. Inhibition of the FGFR family by BAY121 results in the suppression of early microglial reactivity and reduces the neuroinflammatory footprint at later stages, which ultimately leads to an improved preservation of neuronal and synaptic integrity in the site of injury.

TBI results in the simultaneous activation of multiple RTK families, often with distinct temporal dinamics (19): the activation of HGFR/Met has been shown to lead to a reactive, phagocytic microglial state with detrimental consequences (19), whereas the activation of the ErbB family in inhibitory interneurons controls synaptic plasticity and circuit activity after TBI (27). Likewise, activation of the VEGF-C/VEGFR3 contributes to drive microglial polarization after TBI (28) and multiple members of the Tyro-Axl-Mer RTK family regulate microglial reactivity and phagocytic activity in TBI (29) and other disease conditions (30). Our focused screening, together with the immunohistochemical confirmation, reproduce the activation of Met, ErbB4, EphB2 and ErbB4 previously identified (19, 27) and identify a significant phosphorylation of several members of the FGFR family. The pan FGFR inhibitor BAY121 effectively suppresses FGFR1, FGFR3 and FGFR4, with limited effect on the trauma-related activation of other RTK. A notable exception is the full blockade of the induction of ErbB4 phosphorylation; since ErbB4 is highly involved in synaptic plasticity and stability (31, 32) and it is activated by TBI (27), it is possible that this effect of Rogaratinib, possibly mediated by microglia modulation, may contribute to the maintained of circuit integrity and excitation/inhibition balance. The combined screening and immunohistochemistry data demonstrate that BAY121 is effective and relatively selective in preventing FGFR family receptors activation in microglia when administered 30 min after trauma, i.e. in a therapeutic window.

Interestingly, some degree of FGFR phosphorylation was detected also in sham samples; this suggests that a baseline FGFR activation in microglia may control the physiology of these cells in homeostatic conditions. Multiple FGFR ligands are normally expressed in the brain by neurons, astrocytes and other cells (33) and therefore are posited to control microglia as well. Microglial phagocytosis is involved not only in debris clearing upon injury but also in synaptic pruning (34), and FGF/FGFR may contribute to the regulation of this process as well.

The blockade of FGFR signaling by BAY121, both in acute (single dose) or protracted (3 to 7 days) administration, consistently results in the dampening of the neuroinflammatory response to TBI at the level of microglial reactivity as well as in terms of broad inflammatory proteomic footprint. In particular, whereas TBI elevates the protein level of CD40 and Axl both at 3d and 7d, Rogaratinib downregulates CD40 and Axl at 3dpi. Axl and CD40 expression corresponds to the induction of a reactive microglial state with upregulated phagocytosis, coherent with the tissue debris clearance occurring after injury (35–37) and the suppressed upregulation by Rogaratinib at 3d is compatible with the reduced reactivity of microglia (and better synaptic preservation at later stages). Although CD40 and Axl are still upregulated by TBI at 7dpi, they do not appear in the proteome subset downregulated by Rogaratinib. While the negative effect at 7dpi cannot be attributed only to biological effects without further investigations (heterogeneity of TBI mice increases at 7dpi -see (19) potentially increasing the number of false negatives), it could be possible to speculate that an escape from Rogaratinib effect, either in microglia or in infiltrating immune cells, could take place at 7dpi and account for the lack of downregulation in CD40 and Axl.

Interestingly, FGFR family members are expressed not only on microglia but also on a number of immune cells, suggesting their broad contribution to immunity and inflammation: FGFR2 mediates chemotaxis in neutrophils (38), and FGFR1 is expressed in T-cells (39, 40) as well as on macrophages and lymphocytes in lupus nephritis (41). At brain level, FGFR signaling contributes to microglial reactivity to bacterial products by inducing pro-inflammatory cytokines (42) and a small-molecule FGFR inhibitor reduces the infiltration of lymphocytes and activated macrophages, as well as the production of pro-inflammatory cytokines, in the EAE model (22). Thus, our data are coherent with a role of the FGFR family in modulating the overall neuroinflammatory cascade and cytokine response to injury, including microglial reactivity. Interestingly, FGFR expressed on oligodendrocytes also contribute to the regulation of neuroinflammatory cascades in EAE: conditional deletion of FGFR1 or FGFR2 from oligodendrocytes results in decreased microglial reactivity and lymphocytes infiltration in EAE as well as in reduced levels of pro-inflammatory cytokines (43, 44); thus, FGFR blockade may immunomodulatory effects through additional cell types. Although in our model blockade of FGFR resulted in reduced neuroinflammation and enhanced synaptic and neuronal preservation in our model, anti-inflammatory effects of FGFR have been also reported: systemic administration of FGF21 reduces the inflammatory response in a stroke model (45) and intranasal administration of FGF20 reduced blood-brain-barrier impairment in severe TBI (46). Thus, the net effect of FGFR activation may depend on the complex interplay of the inflammatory context and of the mix of FGFR ligands available (42). To this respect, multiple RTK are activated simultaneously in mild TBI [**Figure 1**, (19)]; blockade of FGFR does not affect other RTK also involved in regulation of microglial reactivity (such as HGFR/Met), although a similar anti-inflammatory effect is observed. This may imply a functional redundance of RTK regulation of reactive microglia, or may suggest that the ultimate phenotype is dependent on the combinatorial activation or one or more RTK, which would enable the fine-tuning of the response to the specific conditions. Hence, FGFR signaling may be ultimately pro- or anti-inflammatory depending on the ensemble of RTK activation and the context conditions.

Histological readouts at 7 dpi demonstrate that protracted BAY121 administration results in a reduced loss of excitatory and inhibitory synapses and in the overall preservation of neuronal survival. The proteomic analysis highlights the lack of inflammatory mediators in BAY121-treated samples (still present in the T-Veh samples) and the downregulation of two clusters of proteins involved in proteasome regulation, Golgi function, ribosome biosynthesis and a third cluster of proteases and protease inhibitors. The reduced microglial reactivity (reduced DAM-like phenotype and decreased CD68 expression) may contribute to the preservation of synaptic integrity, since microglia mediates synaptic elimination in several disease settings (47, 48); the downregulation in multiple proteases and protease inhibitors enriched in phagocytes, such as Cathepsins and Serpins, is compatible with this model. In addition, the downregulation of proteasome-regulators and in trafficking proteins may point toward a more limited synaptic de-stabilization and reduced degradation of synaptic components [many of which, are proteasome-dependent: (49–51)]. It must be stressed that FGF/FGFR themselves are regulator of synaptogenesis and synaptic stability (52, 53) and, although we observed a positive impact on synaptic preservation, this outcome may be the net consequence of effects on microglial as well as on other cells types.

A few limitations of the present work are worth addressing. BAY121 is a small-molecule pan FGFR inhibitor; however, the possibility of small-molecule tyrosine kinase inhibitors having additional targets (26, 54), possibly contributing to their biological effect, cannot be fully discounted; to date, our array RTK screening does not demonstrate substantial inhibition besides the FGFR family. In particular, BAY121 (Rogaratinib) is reported to have IC_50_ in the low-nanomolar range for FGFRs (26) but an IC_50_ in the sub-micromolar range for CSF1R. Although the IC50 is more than 100-fold larger for CSF1R than for FGFRs, given the relevance of CSF1R in microglia physiology and pathophysiology, the relative contribution of this low-affinity target to the overall Rogaratib efficacy remains to be fully elucidated.

Furthermore, the effect of systemic administration of a pan-FGFR inhibitor may be not restricted only to microglia but may involve additional players in the CNS [e.g., neurons, oligodendrocytes, immune cells; (33, 53, 55)] although at least in the acute phase, phosphorylation of FGFR1 and 3 is largely restricted to Iba1+ cells. Finally, FGFR are endowed with highly pleiotropic functions in synaptic stability (53), axonal guidance (56) and myelination (57) and the full spectrum of the beneficial and detrimental consequences of FGFR inhibition in acute TBI are not fully elucidated.

## Conclusion

Our findings provide a proof-of-concept of the translational value of targeting the FGFR in acute TBI for the modulation of early microglial reactivity and enhanced preservation of neuronal and synaptic integrity. Recently, rogaratinib-HCl entered clinical trials (58, 59) and three FGFR inhibitors (pemigatinib, futibatinib, and infigratinib) have been approved for human use in the therapy of several gastrointestinal, urologic or haematopoietic neoplasms (25, 60). Although their chronic administration is not devoid of side effects (60), acute or short-term administration may maximize their immunomodulatory and anti-inflammatory effects without interfering with tissue regeneration. In this context, one may envision the administration of FGFR inhibitors in patients with severe neurotrauma and evidence of intense neuroinflammatory responses or synaptic damage (e.g., using synaptic damage biomarkers; 61), in order to limit mcroglia-driven damage. Our findings support the investigation of the repurposing of FGFR inhibitors in this direction. Preliminary to in-human applications, the long-term impact of acute or subacute FGFR inhibitors should be studied using behavioural readouts (e.g., motor and cognitive readouts), together with their impact on astrocyte prolieration, scar formation and oligodendrocyte survival. Given the multiple cellular subpopulations affected by FGFR in the brain, the use of a panel of peripheral biomarkers may contribute to disentangle the protective effects of FGFR inhibitors.

## Materials and methods

### Animals

All experimental procedures were performed in compliance with animal protocols approved by the local veterinary and animal experimentation committee at University Ulm and by the Regierungspraesidium Tubingen under the license no. 1370. B6SJL male mice aged between p60–p90 days were used throughout the study.

### Pharmacological treatment

Rogaratinib (BAY 1213802) was obtained by Bayer Pharma and was administered by oral gavage (200µl) dissolved into the following vehicle: 10% ethanol, 40% Solutol^®^ HS 15, 50% water (26); vehicle alone was administered as control. The dose of 25mg/kg (26) was used throughout this study.

### Traumatic brain injury procedure

Modified closed, blunt weight drop model Traumatic Brain Injury (TBI) was performed as previously reported (19). For all procedures, mice were anesthetized with sevoflurane (2-4% in 96% O2) and were subcutaneously injected with buprenorphine (0.1mg/kg; 1 dose/day) as a pre- and postoperative analgesic. The scalp was shaved and eye ointment was applied preoperatively to protect the cornea. Scalp skin was then incised on the midline to expose the skull and the animals were positioned in the weight-drop apparatus in which the head was secured to a holding frame. Using the 3-axis mobile platform in the apparatus, the impactor was positioned to the coordinates of the injection site (From bregma ≈ x = +3.0mm, y = − 2.0mm, z = 0.0mm). TBI was delivered by dropping a weight of 120g from a height of 45 cm. A mechanical stop prevented a skull displacement (by the impactor) larger than 2.5 mm, in order to keep the brain damage comparable. Apnea time was monitored after injury. The Neurological Severity Score (NSS) was assessed after 3h, 1 dpi and at 7 dpi and never exceeded the score 1 for any mouse. As such, no animal met the criteria for early sacrifice. Mice were checked every 2 hours on the day of trauma. Effort was made to minimize the suffering of animals and reduce the number of animals used. The contralateral hemisphere was used as control samples throughout the study.

### Neurological severity score measurement

Throughout all animal experiments the NSS (62) was measured at 3h, 1h and 7d, depending on sacrificial time of the individual mouse. The NSS is comprised of a total of 10 individual tasks mice were subjugated to each timepoint the NSS was measured with a 10 to 30 seconds break inbetween each individual test. The tests include an arena escape within 3min, mono-/hemiparesis, straight walking, search behaviour, startle reflex, balancing on a) a 7mm wide angular beam and b) a 5mm wide round beam and finally a beam walk test with a lenght of 30cm and a with of a) 3cm, b) 2cm or c) 1cm. Points were awarded when mice could not fulfill an individual task, which then were summed up into the total NSS score. The total NSS score for all animals sacrificed as part of the publication are reported in **Supplementary Table S1**.

### Immunohistochemistry

Brain samples were processed as previously described (7, 19). Briefly, mice were sacrificed by trans-cardial perfusion with 4% PFA in PBS, and brains were dissected and postfixed in 4% PFA overnight. Brains were then transferred to 30% Sucrose for 2 days, after which the samples were embedded in OCT (Tissue Tek, Sakura, Germany). 40-micron sections were cut with a cryostat (Leica CM 1950 AG Protect cryostat). Sections spanning the injury site were selected and blocked (3% BSA, 0.3% Triton in 1x PBS) for 2h at room temperature, followed by incubation for 48h (Iba1, NeuN, VGAT, VGLUT1) or 72h (Iba1, phosphoFGFR1, phosphoFGFR3), at 4°C with primary antibodies diluted in blocking buffer. Identifiers of the antibody used and dilutions are reported in **Supplementary Table S2**. Sections were washed 3x 30 min with PBS and incubated for 2h at RT with secondary antibodies diluted in blocking buffer. The sections were washed with PBS and mounted using Prolong Gold Antifade Mounting Medium (Invitrogen, Germany). A list of the antibodies used in this study can be found in **Supplementary Table S2**.

### Image acquisition and analysis

Confocal images were acquired in 1024 x 1024 pixel and 12-bit format, with a Leica DMi8 inverted microscope, equipped with an ACS APO 40x oil objective. Parameters were set to obtain the optimal signals from the stained antibody or mRNA and at the same time avoiding saturation. All fluorescent channels were acquired independently, to avoid cross-bleed. 3-4 sections spanning the core and perilesional area of the impact site were imaged of each mouse. For each image a tile scan was set up consisting of x by x tiles spanning the injury location.

For image analysis, stacks were collapsed in maximum intensity projection pictures and mean gray value or cell density per fixed region of interest (ROI) was measured. For quantification, we considered a 200µm x 200µm ROI centered on the axis of the injury site.

Synaptic density was detected after producing a mosaic image corresponding to 6 x 6 single optical sections (acquired with a 63x oil objective) with 1 μm optical section thickness. Each cortical section was imaged at a fixed depth 5-10µm inside the section, the composite image was positioned so that an uninterrupted coverage of the impact site with perilesional area was acquired. Quantification of density of synapses was performed using the IMARIS software (Bitplane AG, Zurich, CH) as previously described (63). For the quantification of the histological parameters, we considered a 450µm x 450µm (microglial imaging) or 200 µm × 200 µm (synapses count) ROI centered on the axis of the injury site (“core”) into the cerebral cortex (layer II/III). To measure the intensity of immunolabeling in microglia, we first subtracted the background intensity (Rolling Ball algorithm, ball diameter = 30), then individual cells were traced using the Iba1 immunolabeling as mask and the intensity of individual cell was logged. To determine the percentage of positive cells, for FGFR1 we considered all cells with average intensity higher than 400 arbitrary units (after background subtraction) and for FGFR3 we considered all cells with intensity higher than 100 arbitrary units (after background subtraction) counted every mean intensity above 400 (after subtracting the background mean value) as a positive. The number of positive cells was then divided by the number of total Iba1+ cells in the ROI under consideration.

### Phospho RTK array processing

Proteome Profiler Mouse Phospho-RTK Array Kit (R&D Systems, Minneapolis) was used to determine the Phospho RTK activation pattern. The nitrocellulose membrane arrays provided in the kit were based on sandwich immunoassay and processed according to manufacturer’s instructions. Briefly, membranes spotted with the anti-RTK antibody were blocked in Array buffer 1 for 1h at RT. 160 ug of protein extracted from cortical samples was diluted in 1.5mL Array buffer 1 overnight at 4°C. After washing, arrays were incubated for 2 hours at RT with Anti-Phospho-Tyrosine-HRP Detection Antibody, diluted to 1:5000 in 1X Array Buffer 2. After final washing steps, HRP detection was performed by adding 1 ml Clarity Max™ Western ECL Blotting Substrates from Bio-Rad. Arrays were imaged using BioRad X-ray imager and quantified using ImageJ. ROI was drawn on each antibody spot with a constant diameter and mean gray value was recorded. Further analysis was performed using R software.

### Proteome array processing

200 ug of protein extracted from cortical samples was loaded to the arrays. The arrays were processed according to the manufacturer’s instructions. Briefly, the sample was biotinylated and was mixed with reaction stop reagent and incubated for 30 mins at RT. The glass arrays were incubated in a blocking solution for 45 minutes and washed. After adding a biotinylated sample to the coupling solution, arrays were incubated for 2 hours at RT and washed. The arrays were incubated with a detection buffer with Cy5-streptavidin (ThermoFisher) at 1:1000 for 20 minutes at RT. The washing steps were repeated as described in the manufacturer’s instructions. After removing excess ddH20 from the slides, the arrays were dried. The arrays were imaged using a GenePix 4000B array scanner (Molecular Devices, LLC) and the image analysis was performed using GenePix Pro Software v7 (Molecular Devices, LLC). The settings for the analysis were kept constant in all cases. The GAL file was loaded in the software and the ROIs were adjusted on the protein spots. Each intensity on F635 was recorded and GPR files were saved. Further analysis was performed using R software.

### Protein-protein interaction network and hub genes

We constructed PPI networks to analyze the functional interactions among differentially expressed proteins, using Search Tool for the Retrieval of Interacting Genes/Proteins (STRING: http://www.stringdb.org) (64) and visualized the networks using Cytoscape (https://cytoscape.org/) (65).

### Array bioinformatic analysis

Chemiluminescence signal for each spot was logged after microarray image analysis. The raw intensity values for each receptor/protein were recorded automatedly via image recorder software. The raw data files were loaded in R software and the dataset for each array was preliminary subjected to quality control assessment (QCA), outlier identification, data distribution, intra-array and inter-array normalization. Normalized data for each array was subjected to principal component analysis (PCA) to display group-based clustering. Confidence ellipses (assuming multivariate normal distribution) with the first two principal components were plotted to validate further analysis. Modified linear modeling-based analysis was then applied to the data to identify significant increase or decrease in phosphorylation or protein level. For protein array analyses, the code has been made publicly available on open-access GitHub repository PROTEAS (19).

### Statistics

Statistical analysis was performed with the GraphPad Prism software suite. Normality was routinely checked using the Shapiro-Wilk test and equal variance was tested using the Brown-Forsythe test. Two-way ANOVA with Tukey correction was used for four group comparisons to examine statistical significance. Protein array analysis was performed using R software using the PROTEAS algorythm (previously published; 19), available on GitHub (https://github.com/Rida-Rehman/PROTEAS), with FDR correction at 0.05. Error bars represent standard deviation (SD), unless indicated otherwise. Statistical significance was set at P < 0.05.

## Data Availability

The raw data supporting the conclusions of this article will be made available by the authors, without undue reservation.

## Supplementary material

Supplementary Figure 1 Comparison of TBI- Veh and TBI- BAY121 at 3d post injury. **(A)** Protein-protein interaction (PPI) of significantly upregulated (red) and down regulated (blue) proteins. Color distribution is based on fold change (FC). **(B, C)** Gene ontology (GO) analysis and for **(B)** down regulated proteins and **(C)** upregulated proteins.

Supplementary Figure 2 Comparison of TBI- Veh and TBI- BAY121 at 7d post injury. **(A)** Protein-protein interaction (PPI) of significantly upregulated (red) and down regulated (blue) proteins. Color distribution is based on fold change (FC). **(B)** Gene ontology analysis for down regulated proteins.

Supplementary Table 1 NSS scoring. This Excel file reports all the individual NSS scores calculated for each mouse sacrificed as part of this publication.
