# Supplementary figures and images for: The FGFR inhibitor Rogaratinib reduces microglia reactivity and synaptic loss in TBI
Source: Front Immunol. 2024 Nov 20;15:1443940. doi: 10.3389/fimmu.2024.1443940 (PMC11614719; doi:10.3389/fimmu.2024.1443940)

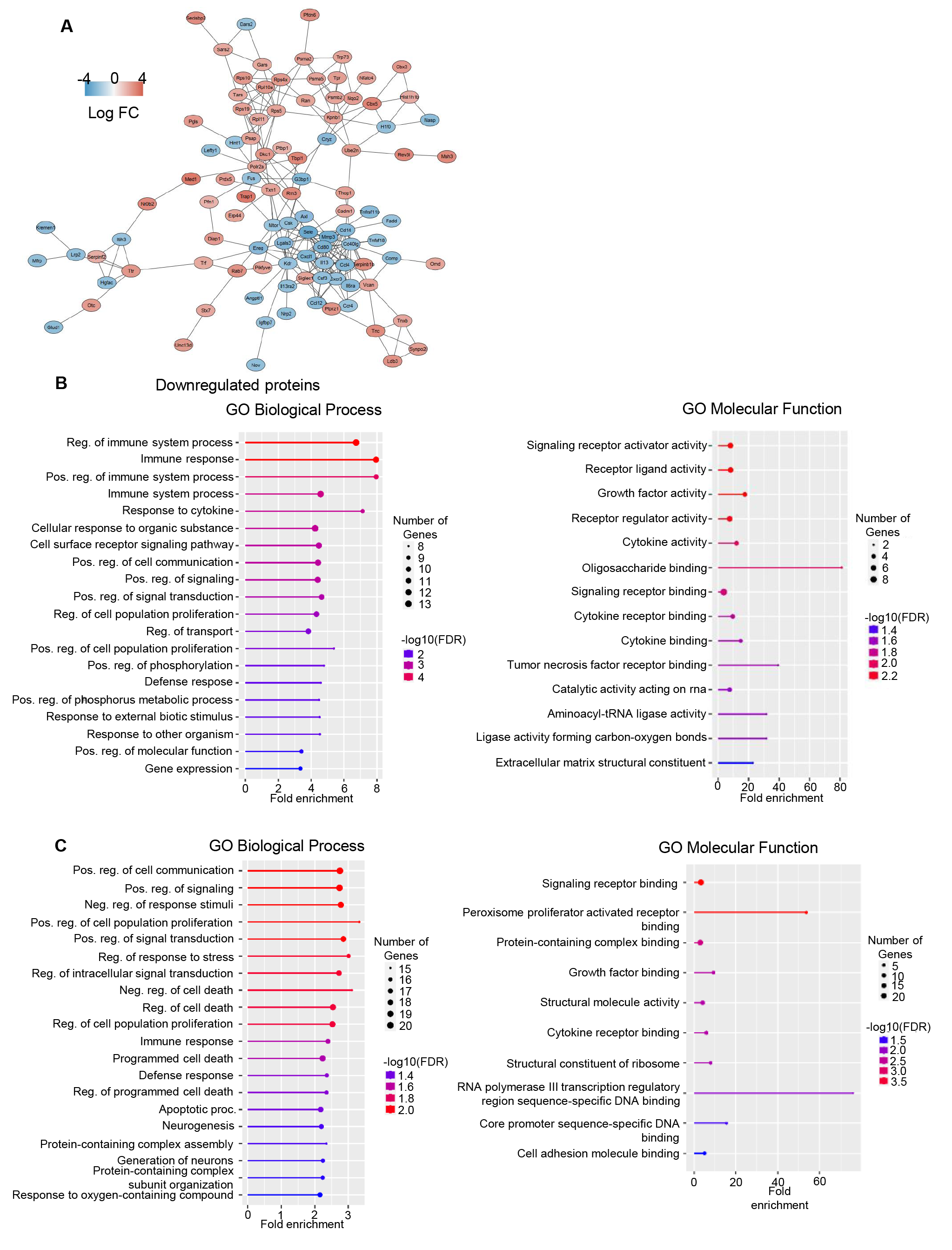

Supplement: Supplementary Figure 1 — Comparison of TBI- Veh and TBI- BAY121 at 3d post injury. (A) Protein-protein interaction (PPI) of significantly upregulated (red) and down regulated (blue) proteins. Color distribution is based on fold change (FC). (B, C) Gene ontology (GO) analysis and for (B) down regulated proteins and (C) upregulated proteins. [file Image1.tif]

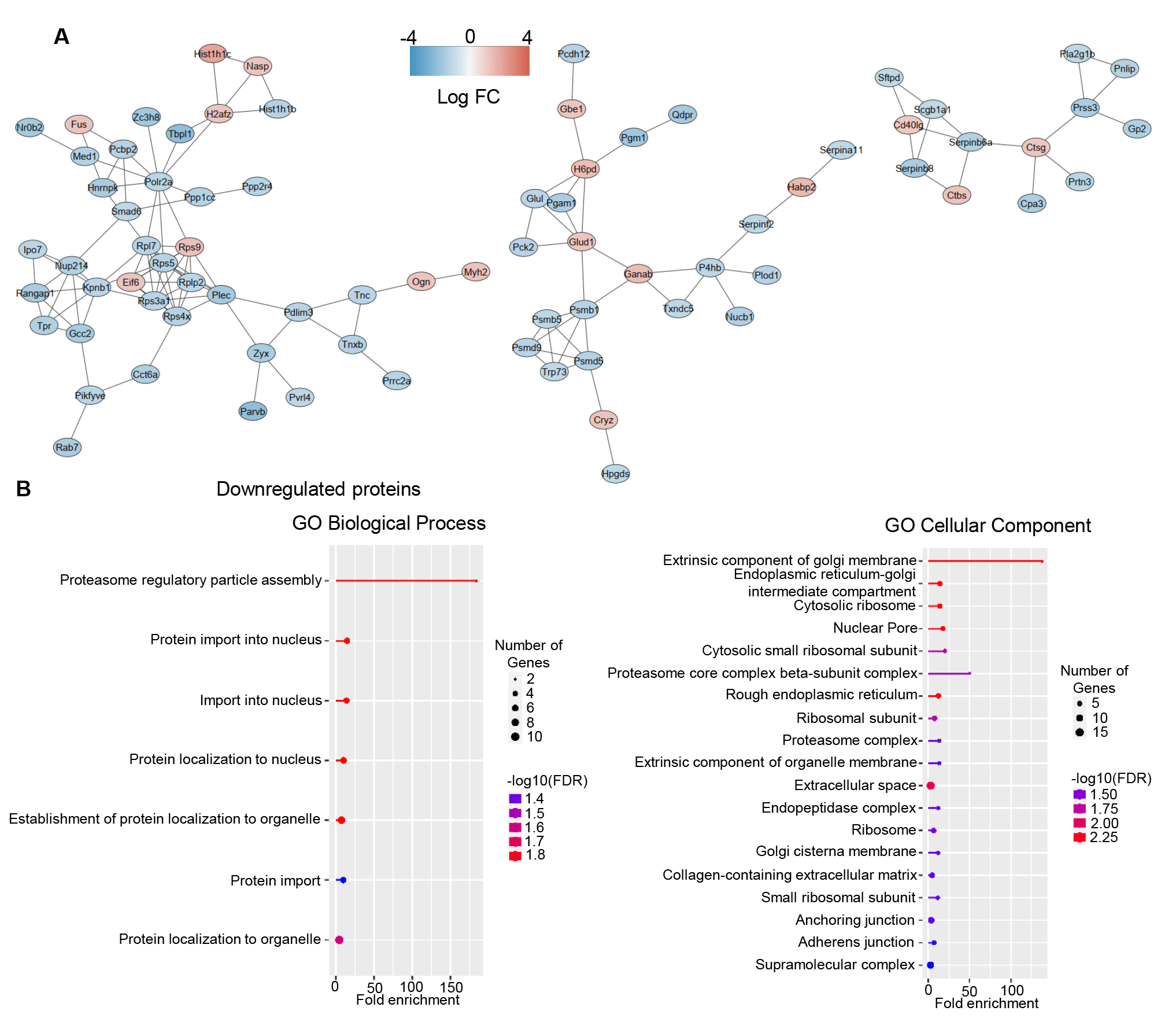

Supplement: Supplementary Figure 2 — Comparison of TBI- Veh and TBI- BAY121 at 7d post injury. (A) Protein-protein interaction (PPI) of significantly upregulated (red) and down regulated (blue) proteins. Color distribution is based on fold change (FC). (B) Gene ontology analysis for down regulated proteins. [file Image2.tif]
