# Supplementary material for: The FGFR inhibitor Rogaratinib reduces microglia reactivity and synaptic loss in TBI
Source: Front Immunol. 2024 Nov 20;15:1443940. doi: 10.3389/fimmu.2024.1443940 (PMC11614719; doi:10.3389/fimmu.2024.1443940)
Supplement: Supplementary file 4 [file Table2.docx]

**Supplementary Table 2. Detailed Antibody list.**

This list contains all antibody used throughout the publication together with their official name, source and identifier and the conditions of use. See also Methods.

| **Reagent** | **Dilution** | **Source** | **Catalogue number** | **RRID** |
| --- | --- | --- | --- | --- |
| DAPI |  | Thermo Fischer | 62247 |  |
| Guinea pig anti-IBA1 | 1:250 | Synaptic Systems | 234 004 | AB_2493179 |
| Mouse anti-NeuN | 1:300 | Millipore | MAB377 | AB_2298772 |
| Rabbit anti-pFGFR1 | 1:500 | Abcam | ab59194 | AB_941585 |
| Rabbit anti-pFGFR3 | 1:200 | Abcam | ab155960 | AB_3095625 |
| Guinea pig anti-VGLUT1 | 1:500 | Synaptic Systems | 135304 | AB_887878 |
| Guinea pig anti-VGAT | 1:500 | Synaptic Systems | 131004 | AB_887873 |
